# Comparison of pulmonary function test, diffusion capacity, blood gas analysis and CT scan in patients with and without persistent respiratory symptoms following COVID-19

**DOI:** 10.1186/s12890-022-01987-z

**Published:** 2022-05-16

**Authors:** Antje Lehmann, Maximilian Gysan, Dominik Bernitzky, Christina Bal, Helmut Prosch, Sonja Zehetmayer, Ruxandra-Iulia Milos, Karin Vonbank, Wolfgang Pohl, Marco Idzko, Daniela Gompelmann

**Affiliations:** 1 Department of Internal Medicine II, Division of Pulmonology, Medical University of Vienna, Vienna, Austria; 2 Department of Biomedical Imaging and Image-Guided Therapy, Medical University of Vienna, Vienna, Austria; 3 Institute for Medical Statistics, Medical University of Vienna, Vienna, Austria; 4 Department of Pulmonology, Krankenhaus Hietzing, Vienna, Austria

**Keywords:** SARS-CoV2, Long COVID, Diffusion capacity

## Abstract

**Background:**

Long-lasting symptoms following SARS-CoV2-infection have been described in several studies. However, there is only limited knowledge about the ongoing pathophysiology and the association with pathological findings in medical examinations.

**Methods:**

In this post hoc analysis of a prospective trial, 135 patients following COVID-19 were enrolled and grouped with respect to the presence or absence of respiratory ongoing symptoms following COVID-19. Pulmonary function test (PFT), diffusion capacity measurement (TLCO SB and TLCO/VA), blood gas analysis (BGA), laboratory tests and high-resolution computed tomography (HRCT) of patients with persistent respiratory symptoms were compared to those of asymptomatic patients.

**Results:**

In this analysis, 71% (96/135) of all patients (mean age 49 years; range 20–91 years) reported long-lasting symptoms after a median (IQR) of 85 days (60–116) following COVID-19 whereby 57.8% (78/135) complained about persistent pulmonary symptoms. Pathological findings in blood test, PFT, TLCO, BGA and/or HRCT were found in 71.8% and 64.1% of patients with and without long-lasting respiratory symptoms respectively. Patients with persistent respiratory symptoms were significantly younger and presented a significant lower FVC (%), TLC (L), and TLCO SB compared to asymptomatic patients (*p* < 0.05). The multiple logistic regression results in a significant effect of age (p = 0.004) and TLCO SB (*p* = 0.042).

**Conclusion:**

Following COVID-19, a large proportion of patients experience ongoing symptoms, whereby the respiratory symptoms are the predominant complaints. Compared to asymptomatic patients, patients with ongoing symptoms were younger and presented a significant lower FVC, TLC and TLCO SB. The multiple logistic regression demonstrated only a significant association between the TLCO SB as the only PFT parameter and the perceived symptoms.

## Background

The pandemic of the coronavirus disease 2019 (COVID-19) caused by severe acute respiratory syndrome coronavirus type 2 (SARS-CoV-2) is associated with a remarkable worldwide health burden. Over 300 million people are recovering from COVID-19 with profound impact on public health [1]. The ongoing impact of COVID-19 on patients weeks to months following the acute infection has been examined in various studies [2–11]. Different guidelines and definitions related to “long COVID” or “post COVID” are available. In most of them, “ongoing symptomatic COVID-19 (OSC)” is defined as persisting signs and symptoms of COVID-19 from four weeks and up to 12 weeks. “Post-COVID-19 syndrome (PCS)” describes signs and symptoms that develop during or after an infection consistent with COVID-19 and persist beyond 12 weeks [12–14]. Colloquially, "Long COVID" includes both—OSC and PCS. The data related to the incidence and clinical appearance of Long COVID syndrome are still very heterogeneous. Most trials, systematic reviews and meta-analysis reported fatigue (31–58%), sleep disturbances (11–44%), breathlessness (24–40%), psychological distress (12–35%), cough (7–29%) and anosmia-dysgeusia (10–22%) as the most common persistent symptoms following COVID-19 [4, 5, 14]. The evaluation of the risk factors for the occurrence of Long COVID also yielded inconsistent and controversial results [5, 6]. Moreover, only few studies evaluated the association between the perception of symptoms and medically objectifiable sequelae of COVID-19. Initial findings did not show significant association between ongoing respiratory COVID-19 symptoms and pathological pulmonary function tests or radiological abnormalities [5, 6].

These contrary results in various studies with a limited number of patients reflect that the full range of the duration and severity of Long COVID syndrome as well as the risk factors and reasons for long-lasting symptoms are still unknown. Therefore, further trials are required for a better understanding of the clinical appearance of Long COVID syndrome.

The aim of the study is to assess the presence of abnormal pulmonary function tests, radiological and laboratory findings in patients with and without ongoing respiratory symptoms following COVID-19.

## Material and methods

Individuals who were referred to the “Post-COVID-19” outpatient clinic for medial assessment independent of persistent symptoms or severity of COVID-19 were evaluated for participation in an ongoing prospective study that was started in June 2020. This prospective study was performed, in accordance with the provisions of the Declaration of Helsinki, at the Medical University of Vienna, Hietzing Hospital Vienna and Otto-Wagner Hospital, Vienna. The protocol of this trial was approved by the local ethic committee of the university of Vienna (1551/2020) and all patients gave written informed consent. The current study, that presents a post-hoc analysis of this prospective trial, focuses on the association of abnormal pulmonary function tests, radiological and laboratory findings in patients with and without ongoing respiratory symptoms following COVID-19.

### Subject enrolment and assessment of clinical data

Patients aged > 18 years with and without ongoing symptoms who recovered from COVID-19 confirmed by a positive polymerase chain reaction (PCR) within the last 6 months were enrolled consecutively from 06/2020 in an ongoing prospective trial independent of the disease severity. The main exclusion criterion was a pre-existing concomitant lung disease such as chronic obstructive pulmonary disease, asthma or interstitial lung disease. Medical history included the date of PCR test positivity, severity of COVID-19 (need for hospital admission due to COVID-19) and symptoms persistent > 4 weeks following acute COVID-19. All patients underwent pulmonary function test (PFT; including forced vital capacity [FVC], forced expiratory volume in 1 second [FEV_1_], total lung capacity [TLC]), measurement of the diffusion capacity (single-breath transfer factor of the lung for carbon monoxide [TLCO SB] and transfer coefficient [TLCO/VA]). Pathological findings were considered as FVC < 80% predicted, TLC < lower limit of normal (LLN), TLCO SB < 80% predicted and TLCO/VA < 80% predicted. Moreover, all patients received a blood gas analysis (BGA) at room air and laboratory test including inflammatory markers and D-dimer. Fibrinogen > 400 mg/dl, Interleukin-6 (IL-6) ≥ 7 pg/ml, C-reactive protein (CRP) > 0.5 mg/dl and D-dimer > 0.5 µg/ml were considered as pathological findings. A high-resolution computed tomography (HRCT) was performed to assess radiological abnormalities.

As the lung is the predominantly affected organ that is involved during corona-virus infection, the focus in this trial was to evaluate ongoing pulmonary symptoms. Based on the presence of persistent pulmonary symptoms, the patients were divided into two groups—patients with persistent respiratory symptoms and asymptomatic patients. These two groups were compared in order to assess the association of the symptoms with abnormal findings in the clinical and medical examinations. To avoid missing any pathological findings that may be responsible for the ongoing symptoms, only the data of patients who underwent all medical examinations were analysed.

### Statistical analysis

For continuous variables mean and standard deviation for each group and a t-test to compare means is calculated. Only for IL6, CRP, and D-Dimer median and interquartile range (IQR) for each group and a Mann–Whitney U-test is calculated due to the skewed distributions of these variables.

For dichotomous variables, absolute and relative frequencies are calculated and a Chi-Square test to compare frequencies between groups or Fisher exact tests for TLC, IL6, and CRP due to small number of events, respectively.

All variables significant in the univariate tests were further considered in a multiple logistic regression analysis with dependent variable symptoms yes/no. Odds ratios (OR), corresponding 95% confidence intervals and *p*-values are reported.

The significance level for the hypothesis tests is set to 0.05, due to the exploratory character of the study, no adjustment for multiple testing is performed. The analyses were performed with R 4.0.3.

## Results

### Subject characteristics and ongoing symptoms

Out of 152 patients who recovered from COVID-19, 135 subjects (male 48.8%, mean age 48.9 ± 15.7 years, range 19–91 years) underwent all medical examinations (PFT, laboratory test, diffusion capacity measurement, blood gas analysis and HRCT) and were enrolled in this analysis. Seventeen patients did not undergo all the medical examinations for several reasons, e.g. refusal of examination, and were excluded from this study.

**Fig. 1:**
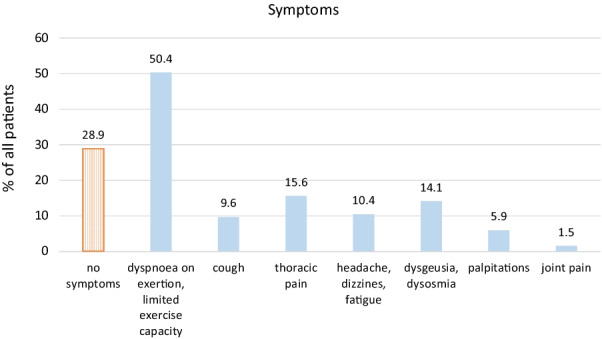
Persistent symptoms in 135 patients after a median of 85 days following COVID-19

**Fig. 2:**
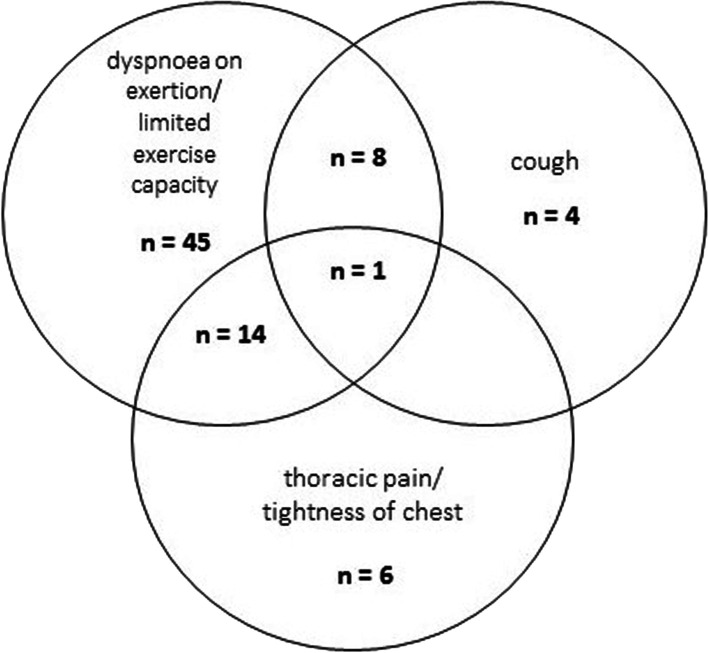
Out of the 135 patients, 78 patients (57.8%) had one or more ongoing respiratory symptoms (dyspnoea on exertion/limited exercise capacity, cough and or thoracic pain/tightness of chest) (Fig. 2)

In this patient cohort, 30 patients (22%) had been hospitalized during acute corona-virus infection. After a median (IQR) of 85 days (60–116) following acute COVID-19, 39 patients out of the 135 patients (28.9%) were symptom-free, whereas 96 patients (71%) reported one or more persistent symptoms (Fig. 1): dyspnoea on exertion and limited exercise capacity in 50.4% (68/135), thoracic pain in 15.6% (21/135), dysgeusia/dysosmia in 14.1% (19/135) and dizziness and headache in 10.4% (14/135) of the subjects. Less frequently, patients reported persistent cough (9.6%), palpitations (5.9%) or joint pain (1.5%). Focussing on respiratory symptoms, 78 out of the 135 patients (57.8%) reported dyspnoea on exertion/limited exercise capacity, cough and/or thoracic pain/tightness of chest (Fig. 2). Results of the pulmonary function test, blood gas analysis and laboratory tests taken at the first visit following COVID-19 are summarized in Table 1.

**Table 1:** Characteristics of 135 patients after a median (IQR) of 85 days (60–116) following COVID-19

	Mean (± STD)	Min	Max
Pulmonary function test			
FVC (L)	4.11 ± 1.11	2	7.27
FVC (%)	95.3 ± 15.3	47	128
FEV1 (L)	3.27 ± 0.93	1.25	5.98
FEV1 (%)	94.5 ± 15.6	50	134
TLC (L)	6.24 ± 1.24	3.58	10.06
TLC (%)	101.4 ± 14.8	51	132
TLCO SB (%)	82.3 ± 16	35.3	120.5
TLCO/VA (%)	93.3 ± 13.5	50.4	131.3
Blood gas analysis			
pO2 (mmHg)	89.3 ± 9.7	61.1	110
pCO2 (mmHg)	38.3 ± 3.9	22	47
Laboratory test			
D-dimer (µg/ml)*	0.28 [0.27, 0.40]	< 0.27	2.56
Fibrinogen (mg/dl)	323 ± 80	201	643
IL-6 (pg/ml)*	1.53 [1.50, 2.34]	< 1.5	65.9
CRP (mg/dl)*	0.28 [0.27, 0.40]	< 0.03	1.33

*Median [IQR]

.

### Symptomatic patients

**Fig. 3:**
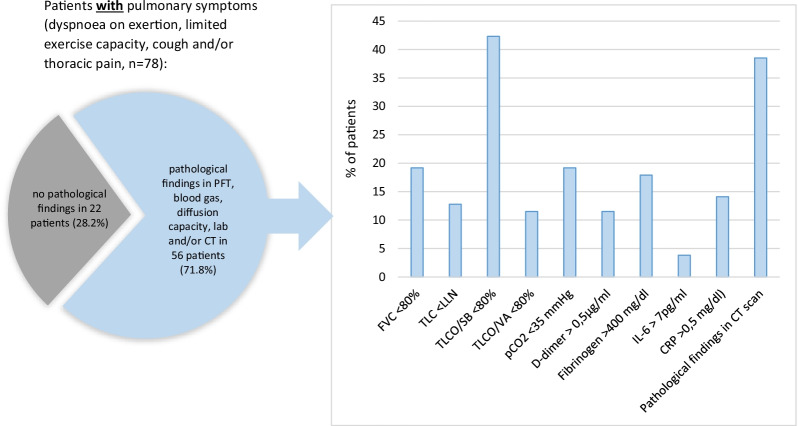
Pathological findings in patients with persistent pulmonary symptoms

Symptomatic patients underwent medical assessment after a median (IQR) of 76.5 days (54-93) following acute COVID-19. In 56 out of the 78 patients (71.8%) with ongoing respiratory symptoms, PFT, TLCO SB, TLCO/VA, BGA, laboratory tests and/or HRCT revealed one or more pathological findings, whereas in 22 patients (28.2%) no pathological results were obtained (Fig. 3). The most common pathological finding was a TLCO SB < 80%, that was observed in 42.3% (Table 2). Moreover, FVC < 80% and TLC < LLN were found in 19.2% and 12.8% of the patients, respectively. The laboratory tests revealed a fibrinogen > 400 mg/dl in 17.9% and an elevated CRP > 0.5 mg/dl and a D-dimer > 0.5 were found in 14.1% and 11.5% respectively. HRCT showed persistent ground-glass opacities and/or consolidations in 38.5% of the symptomatic patients.

**Table 2:** Comparison of characteristics, PFT, BGA, laboratory tests and MDCT in patients with persistent respiratory symptoms and without any ongoing symptoms

	Patients with long lasting respiratory COVID symptoms n = 78	Patients without any long lasting COVID symptoms n = 39	*p*-value
Days following acute COVID-19	76.5 [54; 93]	90.0 [73; 120]	0.019
Age	46.99 ± 15.72	53.08 ± 15.22	0.048
Male (%)	46.2% (36/78)	59% (23/39)	0.266
Hospitalisation in the past due to COVID 19	24.4% (19/78)	23.1 (9/39)	1.000
FVC (L)	4.02 ± 1.08	4.29 ± 1.23	0.224
FVC (%)	91.94 ± 15.66	99.46 ± 13.91	0.012
FVC < 80%	19.2% (15/78)	10.3 (4/39)	0.330
TLC (L)	6.11 ± 1.16	6.61 ± 1.36	0.044
TLC (%)	99.19 ± 15.87	104.69 ± 12.73	0.062
TLC < LLN	12.8% (10/78)	2.6% (1/39)	0.097
TLCO SB (%)	79.87 ± 15.73	87.73 ± 17.50	0.016
TLCO SB < 80%	42.3% (33/78)	33.3% (13/39)	0.462
TLCO/VA (%)	92.75 ± 12.51	96.27 ± 16.74	0.203
TLCO/VA < 80%	11.5% (9/78)	15.4 (6/39)	0.769
pO2 (mmHg)	89.16 ± 9.98	88.78 ± 8.90	0.841
pCO2 (mmHg)	37.92 ± 3.85	39.24 ± 3.67	0.078
pCO2 < 35 mmHg	19.2% (15/78)	12.8% (5/39)	0.543
Fibrinogen (mg/dl)	322.19 ± 88.76	327.82 ± 71.91	0.732
Fibrinogen > 400 mg/dl	17.9 (14/78)	10.3 (4/39)	0.415
IL-6 (pg/ml)*	1.50 [1.50, 2.36]	1.61 [1.50, 2.28]	0.708
IL-6 ≥ 7 pg/ml	3.8% (3/78)	5.1% (2/39)	1.000
CRP (mg/dl)*	0.11 [0.05, 0.24]	0.12 [0.06, 0.19]	0.824
CRP > 0.5 mg/dl	14.1% (11/78)	10.3% (4/39)	0.771
D-dimer (µg/ml)*	0.29 [0.27, 0.36]	0.28 [0.27, 0.50]	0.358
D-dimer > 0.5 µg/ml	11.5% (9/78)	25.6% (10/39)	
Ground glass opacities/consolidation/pulmonary embolism in CT	38.5% (30/78)	28.2% (11/39)	0.448

*Median [IQR]

### Asymptomatic patients

**Fig. 4:**
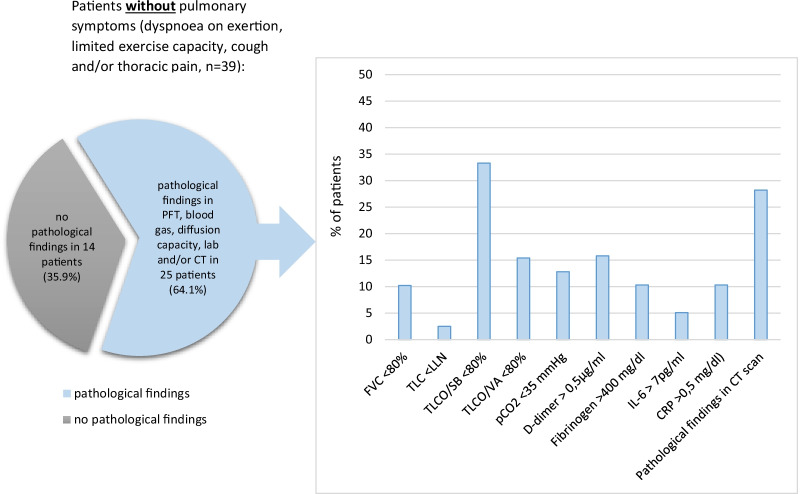
Pathological findings in patients without persistent pulmonary symptoms

Asymptomatic patients underwent medical assessment after a median (IQR) of 90 days (73-120). Within the asymptomatic patient group, pathological results were obtained in 25 out of the 39 patients (64.1%) (Fig. 4). Similar to the symptomatic patients, a reduced TLCO SB was the most common pathological finding followed by persistent ground-glass opacities and/or consolidations in the HRCT (Fig. 4, Table 2).

### Comparison of symptomatic and asymptomatic patients

The percentage of subjects with abnormal results in PFT, TLCO SB, TLVO/VA, BGA, laboratory tests and MDCT did not differ significantly between both groups (Table 2). However, patients with persistent respiratory symptoms following COVID-19 had significantly lower FVC (%), TLC (L) and TLCO SB (%) compared to asymptomatic patients. Moreover, symptomatic patients were significantly younger than patients without any symptoms. The multiple logistic regression resulted in a significant effect of age (*p* = 0.004) and TLCO SB (*p* = 0.042) on the development of persistent respiratory symptoms following COVID-19 (Table 3). The comparison of follow-up periods between symptomatic and asymptomatic patients revealed a significant difference (*p* = 0.019). However, the multiple logistic regression shows that this time variation did not have additional impact on the presence or absence of symptoms (*p* = 0.166).

**Table 3:** Multiple logistic regression analysis

	OR	95% Confidence Interval		*p*-value
Age	0.94	0.91	0.98	0.004
FVC (%)	0.98	0.95	1.02	0.322
TLC (L)	0.80	0.52	1.22	0.235
TLCO SB (%)	0.96	0.92	0.99	0.042
Days following acute COVID-19	0.96	0.92	0.99	0.017

## Discussion

The term “Long COVID” that summarizes OSC and PCS describes a syndrome that can occur after an acute coronavirus infection and is characterised by the persistence of various symptoms > 4 weeks following acute COVID-19. The prevalence of Long COVID has a broad range from 40% [7] to 87% [2] in various studies that may be explained by different follow-up periods and heterogeneous populations. In a meta-analysis Long COVID symptoms were present in more than 60% of patients infected with SARS-CoV-2 [11]. In the current study, the overall incidence of Long COVID after a median of 85 days following the acute infection was found to be 71%. As previously reported [2, 3, 6, 8], the most common symptoms were fatigue and breathlessness.

In our study, the most common pathological finding in the medical examination was a reduced diffusion capacity in 39% of the 117 patients independent of ongoing respiratory symptoms. Zhao et al. also described a reduced diffusion capacity as the most common abnormality in PFT with a significantly reduced TLCO in 16% of the 55 enrolled patients followed by a reduced FVC and FEV_1_ in 10.9% at 3-month follow-up [9]. Huang et al. reported a TLCO value less than 80% in 53% and 35% of patients one month and 6 months following COVID-19 respectively [8, 10]. The impairment of the diffusion capacity indicates an interstitial lung disease that was consistent with our HRCT findings, which revealed pulmonary interstitial changes in 35% of the 117 patients. It was shown that respiratory viral infection induces distinct fibroblast activation that might be responsible for the pulmonary interstitial changes and thus the reduced TLCO SB [8, 15].

In the study of Moreno-Pérez et al. [6], the authors described no association between the perceived symptoms and pathological findings in spirometry and chest radiograph. However, their initial assessment did not include the measurement of diffusion capacity or HRCT that are more sensitive to assess ongoing alterations following COVID-19. In our trial, the PFT, measurements of diffusion capacity, blood gas analysis, laboratory tests and MDCT revealed no abnormal findings in 28% of the symptomatic patients, whereas pathological findings were found in 64% of the asymptomatic patients. These results also suggest at first glance a lack of association between the persistent symptoms and the pathological findings in medical examinations. However, a patient may experience symptoms due to a decrease in FVC, TLC or TLCO SB from their individual baseline without these reaching the lower limit. It must be noted that no PFT taken prior to acute COVID-19 that could serve as a reference was available. However, this seems to play a minor role, as no significant difference between lung function test prior to and following COVID-19 could be found in a study cohort of 80 patients [16]. Comparing symptomatic and asymptomatic patients, the patients with ongoing respiratory symptoms who were younger than the asymptomatic patients had a significantly lower FVC (%), TLC (L) and TLCO SB (%) compared to asymptomatic patients and thus indicating an association between perceived symptoms and pathological findings in medical examinations. Our results are similar to the findings of Cortés-Telles et al. who described a significantly lower FVC, FEV_1_, TLCO in patients with persistent dyspnoea compared to asymptomatic patients following COVID [17].

The multiple logistic regression that we performed in our study resulted in a significant effect of age and TLCO SB on ongoing respiratory symptoms following COVID-19. We found no significant association between the rate of hospitalization during acute COVID-19 and persistent pulmonary sysmptoms. In contrast to our finding, Arnold et al. described an association between the development of persistent respiratory symptoms following COVID-19 and the severity of the acute COVID-19 infection defined as the need of supplementary oxygen, non-invasive or invasive ventilation [18]. More studies are required to evaluate the predictive value of the disease severity on the development of Long COVID.

The limitation of this study includes the missing baseline PFT data prior to COVID-19. Therefore, the impairment of PFT cannot be directly attributed to the coronavirus infection. However, patients with pre-existing lung disease were excluded from this trial, so that it may be assumed that the enrolled patients had normal PFT data prior to the acute virus infection. Other limitations are the relatively small sample size and the short follow-up period of up to three months. Furthermore, it must be noted, that there may by a selection bias as 17 patients who did not undergo all the examinations were excluded from this analysis. The reason why these patients were excluded was to avoid potentially undetected medically objectifiable sequelae that could have led to ongoing symptoms.

## Conclusions

Summarizing, a large proportion of patients experience persistent symptoms following COVID-19, whereby the respiratory symptoms are the predominant complaints. Although the proportion of patients with a TLCO SB < 80% predicted did not differ significantly between the symptomatic and asymptomatic patients, the symptomatic patients presented with a significantly lower TLCO SB. Multiple logistic regression also resulted in a significant effect in TLCO SB and thus indicating an association between an impaired diffusion capacity and the perception of symptoms. Longer follow-up trials in larger populations are needed to better understand the pathophysiology of Long COVID, its outcome and its management.

## Data Availability

All data generated or analysed during this study are included in this published article.

## Abbreviations

BGA: Blood gas analysis
COVID-19: Corona virus disease 2019
CRP: C-reactive protein
FEV_1_: Forced expiratory volume in 1 second
FVC: Forced vital capacity
HRCT: High-resolution computed tomography
PCR: Polymerase chain reaction
SARS-CoV-2: Severe acute respiratory syndrome coronavirus type 2
TLCO SB: Single-breath transfer factor for carbon monoxide
TLCO/VA: Transfer coefficient
TLC: Total lung capacity
